# Assessment of the relative benefits of monotherapy and combination therapy approaches to the treatment of hospital-acquired *Stenotrophomonas maltophilia* pneumonia: a multicenter, observational, real-world study

**DOI:** 10.1186/s13613-023-01144-7

**Published:** 2023-06-06

**Authors:** Liang Chen, Jie Hua, Shujie Hong, Chenyang Yuan, Ruochen Jing, Xuanyu Luo, Yihong Zhu, Le Le, Ziqi Wang, Xiaoli Sun, Xiaopu He

**Affiliations:** 1 Department of Infectious Diseases, Nanjing Lishui People’s Hospital, Zhongda Hospital Lishui Branch, Southeast University, Nanjing, China; 2 Department of Gastroenterology, The First Affiliated Hospital of Nanjing Medical University, Nanjing, China; 3 Department of Clinical Medicine, Nanjing Medical University, Nanjing, China; 4 Department of Geriatric Gastroenterology, The First Affiliated Hospital of Nanjing Medical University, No. 300 Guangzhou Road, Nanjing, 210029 China

**Keywords:** *S. maltophilia*, Hospital-acquired pneumonia, Efficacy, Monotherapy, Combination therapy

## Abstract

**Purpose:**

*Stenotrophomonas maltophilia* is a Gram-negative pathogen that most commonly causes hospital-acquired infections that can be extremely challenging to treat, contributing to underrecognized mortality throughout the world. The relative benefits of monotherapy as compared to combination therapy in patients diagnosed with *S. maltophilia* pneumonia, however, have yet to be established.

**Methods:**

Data from 307 patients diagnosed with *S. maltophilia* hospital-acquired pneumonia (HAP) across four Chinese teaching hospitals from 2016 to 2022 were retrospectively analyzed.

**Results:**

Of the analyzed patients, 55.7% (171/307) were administered combination definitive therapy, with a 30-day all-cause mortality rate of 41.0% (126/307). A propensity score weighting analysis revealed that compared with monotherapy, combination definitive therapy was associated with a comparable 30-day mortality risk in the overall patient cohort (*OR* 1.124*, 95% CI* 0.707–1.786*, P* = 0.622), immunocompetent patients (*OR* 1.349*, 95% CI* 0.712–2.554*, P* = 0.359), and patients with APACHE II scores < 15 (*OR* 2.357*, 95% CI* 0.820–6.677*, P* = 0.111), whereas it was associated with a decreased risk of death in immunocompromised patients (*OR* 0.404*, 95% CI* .170–0.962*, P* = 0.041) and individuals with APACHE II scores ≥ 15 (*OR* 0.494*, 95% CI* 0.256–0.951*, P* = 0.035).

**Conclusion:**

The present data suggest that when treating *S. maltophilia-*HAP, immunocompromised patients and individuals with APACHE II scores ≥ 15 may potentially benefit from combination therapy.

**Supplementary Information:**

The online version contains supplementary material available at 10.1186/s13613-023-01144-7.

## Background

The Gram-negative, aerobic, non-glucose fermenting, motile bacteria *Stenotrophomonas maltophilia* is most frequently identified as a free-living microbe present in humid and aquatic settings, whereas it is not a normal member of the human microflora [1]. It is most frequently regarded as an opportunistic pathogen capable of surviving in humid settings such that it has been isolated from nosocomial sources including shower heads, sink grains, endoscopes, nebulizers, and hemodialysis dialysate samples [2]. While it is generally regarded as a pathogen with low levels of intrinsic virulence, rates of *S. maltophilia* infections have been rising in recent decades, in large part owing to advances in immunocompromised patient care, broad-spectrum antibiotic use, and the application of invasive devices [3, 4]. *S. maltophilia* accounts for 2.29–22.7% of isolated Gram-negative bacteria, and this rate is higher at 2–7% among cancer patients throughout the world. *S. maltophilia* is also the third most frequently isolated non-fermenting Gram-negative pathogen following *Pseudomonas aeruginosa* and *Acinetobacter baumannii* [4].

In susceptible individuals, *S. maltophilia* can cause infections including meningitis, urinary tract infections, bloodstream infections, and pneumonia, with mortality rates from 29 to 70% [5]. This is largely attributable to the broad-spectrum drug resistance exhibited by this opportunistic pathogen, which encodes aminoglycoside modifying enzymes, multidrug efflux pumps, and β-lactamases, in addition to exhibiting low levels of intrinsic permeability [6, 7]. *S. maltophilia* can also acquire drug resistance over the course of treatment. Given these characteristics, *S. maltophilia* has been classified by the World Health Organization as one of the most important nosocomial multidrug-resistant organisms [8]. Based on the results of in vitro susceptibility testing, the antibiotics most often used to treat *S. maltophilia* infections in Chinese hospitals include trimethoprim/sulfamethoxazole (TMP-SMX), fluoroquinolones, and tetracycline derivatives [9]. In patients with hospital-acquired pneumonia (HAP) caused by this pathogen, there is no consensus regarding the optimal interventional approach, with some experts recommending a combination of antibiotics. Clinical data regarding the benefits of such combination treatment, however, remain to be generated such that the value of this interventional strategy has yet to be firmly established.

The present multicenter retrospective analysis was performed in an effort to compare the relative clinical benefits of monotherapy as compared to combination therapy in patients diagnosed with *S. maltophilia-*HAP.

## Materials and methods

### Study design

Patients diagnosed with *S. maltophilia-*HAP were retrospectively identified using medical records from individuals hospitalized across four Chinese teaching hospitals (Additional file 1). Patients were not eligible for inclusion if they were less than 14 years of age or if they died within 48 h after first receiving definitive therapy. A two-level review process was employed for all collected data, with a third investigator serving to resolve any disputes pertaining to the interpretation of these data. Study reporting was performed as per the STROBE guidelines (https://www.equator-network.org/reporting-guidelines/strobe/).

The primary study outcome was all-cause 30-day mortality after the initiation of definitive therapy. Secondary outcomes included the 30-day clinical response, assessed by improvements in patient symptoms according to laboratory tests or physician records, as well as 30-day bacterial eradication assessed by the results of repeat cultures of respiratory tract samples.

This study received approval from the Ethics Committee of Nanjing Lishui People’s Hospital (No. 2022SQ009), with the requirement for informed consent being waived given the retrospective nature of this work.

### Microbiological analyses

MALDI-TOF mass spectrometry (MALDI Biotyper, Bruker Daltonics GmbH, Leipzig, Germany, or Vitek-MS, bioMérieux) or the Vitek 2 platform (bioMérieux, Marcy l’Etoile, France) were used when identifying the microbial isolates in this study. The Vitek 2 system was used to conduct antibiotic susceptibility testing in most cases based on standardized hospital protocols, with the results of such testing being interpreted as per the 2019 CLSI recommendations. Given that these recommendations do not include tigecycline (TGC) or moxifloxacin susceptibility breakpoints for *S. maltophilia, Enterobacteriaceae* susceptibility breakpoints from the US Food and Drug Administration (FDA) were instead used, with a susceptibility TGC minimum inhibitory concentration (MIC) ≤ 2 µg/mL being regarded as indicative of susceptibility [10].

### Study definitions

HAP was defined as cases of pneumonia that were not present at the time of hospitalization and that developed at least 48 h following admission, with pneumonia being defined by the presence of new-onset infectious lung infiltrates, purulent sputum, leukocytosis, reduced oxygen levels, and fever [11]. To ensure the appropriate identification of target pathogens that were consistent with the clinical presentation of evaluated patients, only those pathogens isolated from sterile body fluids, lung tissue, bronchoalveolar lavage fluid, protected specimen brush samples, or qualified lower respiratory tract secretions (> 25 neutrophils per low-power field [LPF], < 10 epithelial cells per LPF, or a neutrophil to epithelial cell ratio > 2.5:1) [11]. Immunocompromised patients were those individuals with a history of diagnosed primary immunodeficiency, HSCT, solid organ transplantation, splenectomy, active malignancies, or human immunodeficiency virus (HIV) Infection with CD4 + T cell counts below 200 cells/mL or a CD4 + T cell percentage below 14% [12]. Septic shock was identified based on the requirement for vasopressor administration to ensure that mean arterial pressure remained ≥ 65 mmHg even with adequate volume resuscitation and serum lactate levels ≥ 2 mmol/L [13]. Empirical treatment was defined as antimicrobial drug administration prior to the completion of susceptibility testing, with appropriate empirical therapy being a regimen containing at least one drug to which the target pathogen was found to be sensitive as a result of in vitro testing [14]. Definitive treatment was defined as any antimicrobial drug treatment administered after obtaining the results of in vitro susceptibility testing [14]. Combination definitive treatment regimens were those consisting of 2 + agents with detected in vitro activity against the target pathogen, whereas monotherapeutic regimens were those including 1 such agent [14].

### Data collection

Patient medical records were retrospectively evaluated to assess demographic characteristics, comorbidities (Additional file 1), microbiological details, administered empirical and definitive antimicrobial treatment regimens, and patient clinical outcomes. Note that these analyses only evaluated first-episode definitive treatment regimens.

### Statistical analysis

The normality of data distributions was assessed with the Kolmogorov–Smirnov test, and normally and non-normally distributed data were reported as means ± standard deviation (SD) and medians (interquartile range), respectively. These data were analyzed using Student’s t-tests and Mann–Whitney U tests, whereas Fisher’s exact test or chi-square tests were employed when analyzing categorical data. A two-tailed P < 0.05 was the significance cut-off for this study. All analyses were performed with SPSS 22.0 (IBM NY, USA), and all eligible patients were included in this study with no power calculations having been conducted.

To control for the potential confounding effects of variables, a propensity score (PS) weighting approach was utilized. PS values were calculated with a multivariate logistic regression model for the likelihood of combination therapy on a per-patient basis. Analyzed covariates included age, sex, body mass index (BMI), participating hospitals, comorbidities (chronic obstructive pulmonary disease, cardiovascular disease, chronic liver disease, cerebrovascular disease, chronic kidney disease, diabetes mellitus, asthma), immunocompromised status, APACHE II scores, coinfection, carbapenem-resistant organism coinfection, secondary bacteremia, septic shock, appropriate empirical treatment, noninvasive mechanical ventilation, invasive mechanical ventilation, vasopressor use, intensive care unit (ICU) admission, and days between HAP onset and definitive treatment. Weighted PS (WPS) values were calculated as: WPS = PT/PS for patients administrated combination therapy, and WPS = (1-PT)/(1-PS) for the patients administered monotherapy, with PT corresponding to the overall proportion of patients in this study that were administered combination therapy [15]. After controlling for these WPS values, the associations between monotherapy or combination definitive therapy and patient outcomes were evaluated.

Univariate regression analysis was first used to evaluate the baseline characteristics in surviving and deceased patients. All factors were found to be significant (*P* < 0,01) and were thus included in a multivariate backward stepwise logistic regression analysis aimed at identifying independent predictors of 30-day mortality. The relative benefits of combination or monotherapy treatment were then compared, using the identified independent risk factors as confounding variables in the multivariate analysis.

## Results

### Patient characteristics

**Fig. 1:**
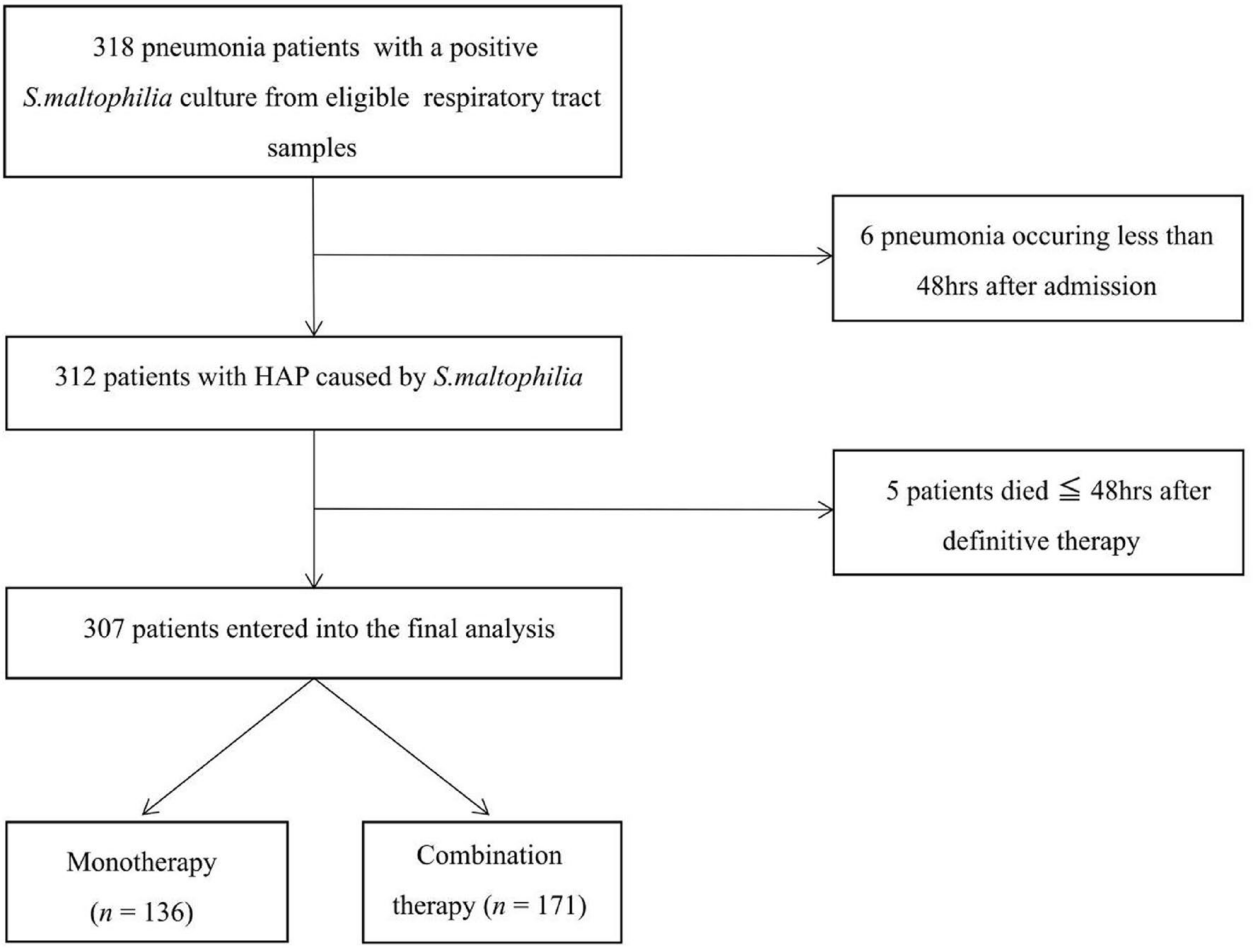
Screening algorithm of patients with *S. maltophilia*-HAP. Figure legend Of 318 identified S. maltophilia-HAP patients diagnosed based on positive culture results for eligible respiratory tract specimens, 307 non-duplicate patients were ultimately enrolled in the final study

Of 318 identified patients with *S. maltophilia-*HAP diagnosed based on positive culture results for eligible respiratory tract specimens, 307 remained following the exclusion of duplicates. These included 136 and 171 patients that, respectively, received monotherapy and combination definitive therapy (Fig. 1). For full details regarding the antimicrobial treatment regimens provided to these patients, see Additional file 1.

These patients were 75.6% male (232/307), with a median age of 65.0 years. The most common comorbid conditions in this patient cohort included cardiovascular disease (57.3%, 176/307), diabetes mellitus (36.8%, 113/307), and cerebrovascular disease (17.9%, 55/307). In addition, 38.1% (117/307) of these patients exhibited factors consistent with immunocompromised status. At baseline, these patients exhibited a median APACHE II score of 17.0, and 17.9% (55/307) experienced septic shock, while 65.8% 202/307) and 50.5% (155/307), respectively, received noninvasive and invasive mechanical ventilation. Appropriate empirical therapy was provided to just 12.2% (37/307) of patients (37 patients received fluoroquinolone treatment and 2 patients were treated with tigecycline), with 75.9% (233/307) of patients having been admitted to the ICU and a 30-day mortality rate of 41.0% (126/307) (Table 1).

**Table 1:** Comparison of clinical characteristics and outcomes between patients receiving combination therapy and monotherapy

Variable	Total (*n* = 307)	Combination therapy (*n* = 171)	Monotherapy (*n* = 136)	*P*-value
Age (years, median, IQR)	65.0 (53.0, 75.0)	68.0 (56.0, 78.0)	61.0 (49.0, 72.0)	0.001
Male (*n*, %)	232 (75.6)	130 (76.0)	102 (75.0)	0.866
BMI (kg/m^2^, mean ± SD)	23.4 ± 8.4	22.9 ± 2.6	24.7 ± 12.2	0.132
Participating hospital				
1	105 (34.2)	65 (38.0)	40 (29.4)	0.115
2	81 (26.4)	46 (26.9)	35 (25.7)	0.818
3	54 (17.6)	28 (16.4)	26 (19.1)	0.531
4	67 (21.8)	32 (18.7)	35 (25.7)	0.139
Comorbidities (*n*, %)				
Cardiovascular disease	176 (57.3)	104 (60.8)	72 (52.9)	0.166
Diabetes mellitus	113 (36.8)	74 (43.3)	39 (28.7)	0.008
Cerebrovascular disease	91 (29.6)	60 (35.1)	31 (22.8)	0.019
Chronic kidney disease	55 (17.9)	37 (21.6)	18 (13.2)	0.057
COPD	52 (16.9)	31 (18.1)	21 (15.4)	0.533
Chronic liver disease	27 (8.8)	15 (8.8)	12 (8.8)	0.987
Asthma	24 (7.8)	13 (7.6)	11 (8.1)	0.875
Immunocompromised status	117 (38.1)	77 (45.0)	40 (29.4)	0.005
Baseline clinical features and severity				
Leukocyte counts (× 10^9^/L)	11.1 ± 7.9	11.6 ± 9.5	10.3 ± 4.5	0.160
PCT < 2 ng/dL (*n*, %)	110 (35.8)	61 (35.7)	49 (36.0)	0.948
PO_2_/FiO_2_ < 300 mmHg (*n*, %)	44 (14.3)	21 (12.3)	23 (16.9)	0.250
APACHE II score (median, IQR)	17.0 (13.0, 22.0)	19.0 (14.0, 23.0)	14.0 (12.0, 19.0)	< 0.001
Coinfection (*n*, %)	150 (48.9)	91 (53.2)	59 (43.4)	0.087
With other CRO (*n*, %)	76 (24.8)	40 (23.4)	36 (26.5)	0.535
Complications and Management				
Secondary bacteremia (*n*, %)	14 (4.6)	6 (3.5)	8 (5.9)	0.322
Septic shock (*n*, %)	55 (17.9)	42 (24.6)	13 (9.6)	0.001
Appropriate empirical therapy (*n*, %)	37 (12.2)	23 (13.5)	14 (10.3)	0.399
Noninvasive mechanical ventilation (*n*, %)	202 (65.8)	116 (67.8)	86 (63.2)	0.399
Invasive mechanical ventilation (*n*, %)	155 (50.5)	103 (60.2)	52 (38.2)	< 0.001
Vasopressor use (*n*, %)	18 (5.9)	12 (7.0)	6 (4.4)	0.334
ICU admission (*n*, %)	233 (75.9)	136 (79.5)	97 (71.3)	0.095
Days from illness onset to definitive therapy (median, IQR)	5.0 (4.0, 6.0)	5.0 (4.0, 6.0)	5.0 (4.0, 6.0)	0.504
Outcomes				
30-day mortality	126 (41.0)	72 (42.1)	54 (39.7)	0.671
30-day clinical response	167 (54.4)	93 (54.4)	74 (54.4)	0.996
30-day microbiology eradiction	131/272 (48.2)	74/152 (48.7)	57/120 (47.5)	0.846

*IQR* interquartile range; *SD* standard deviation; *COPD*: chronic obstructive pulmonary disease. *BMI* body mass index; *PCT* procalcitonin; *PO*_*2*_*/FiO*_*2*_ arterial pressure of oxygen/fraction of inspiration oxygen; *APACHE* Acute Physiology and Chronic Health Evaluation; CRO: carbapenem-resistant organism; *ICU* intensive care unit. *HAP* hospital-acquired pneumonia. Immunocompromised status included primary immune deficiency diseases, active malignancy, HIV infection with a CD4 T-lymphocyte count < 200 cells/mL or percentage < 14%, immunosuppressive therapy, solid organ transplantation, hematopoietic stem cell transplantation, splenectomy

### The overall association between definitive treatment regimen and patient outcomes via propensity score weighting analysis

Compared to those patients who underwent monotherapeutic treatment, those that received combination definitive therapy tended to be older (median, 68.0 vs 61.0 years, *P* = 0.001), were more likely to have diabetes mellitus (43.3% vs 28.7%, *P* = 0.008), cerebrovascular disease (35.1% vs 22.8%, *P* = 0.019), and to be immunocompromised (45.0% vs 29.4%, *P* = 0.005). Patients in the combination therapy group presented with higher APACHE II scores (median, 19.0 vs 14.0, *P* < 0.001), were more likely to develop septic shock (24.6% vs 9.6%, *P* = 0.001), and had a higher chance of undergoing invasive mechanical ventilation as compared to patients in the monotherapy group (60.2% vs 38.2%, *P* < 0.001). The all-cause 30-day mortality (42.1% vs 39.7%, *p* = 0.671), 30-day clinical response (54.4% vs 54.4%, *p* = 0.996), and 30-day microbial eradication (48.7% vs 47.5%, *p* = 0.846) were similar between the two groups. (Table 1).

**Fig. 2:**
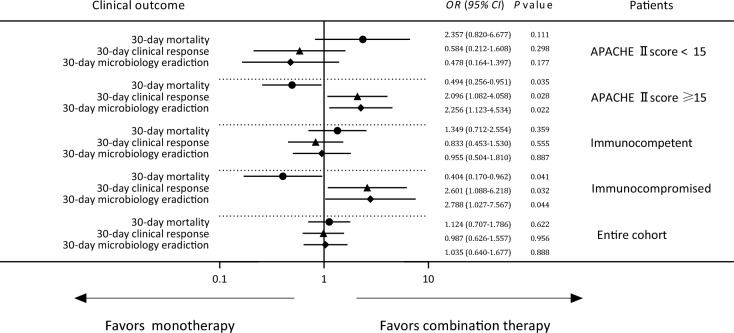
Forrest plot of the impact of monotherapy and combination therapy on the clinical outcomes of patients with *S. maltophilia*-HAP by treatment propensity score analysis. Figure legend Propensity score analyses indicated that in the overall patient cohort, patients with APACHE II scores < 15, and immunocompetent individuals, combination treatment was associated with comparable 30-day mortality, clinical response, and microbiologic eradication outcomes, whereas it was associated with reduced 30-day mortality risk, higher 30-day clinical response rates, and improved 30-day microbiologic eradication outcomes among individuals with APACHE II scores ≥ 15 and immunocompromised individuals

**Fig. 3:**
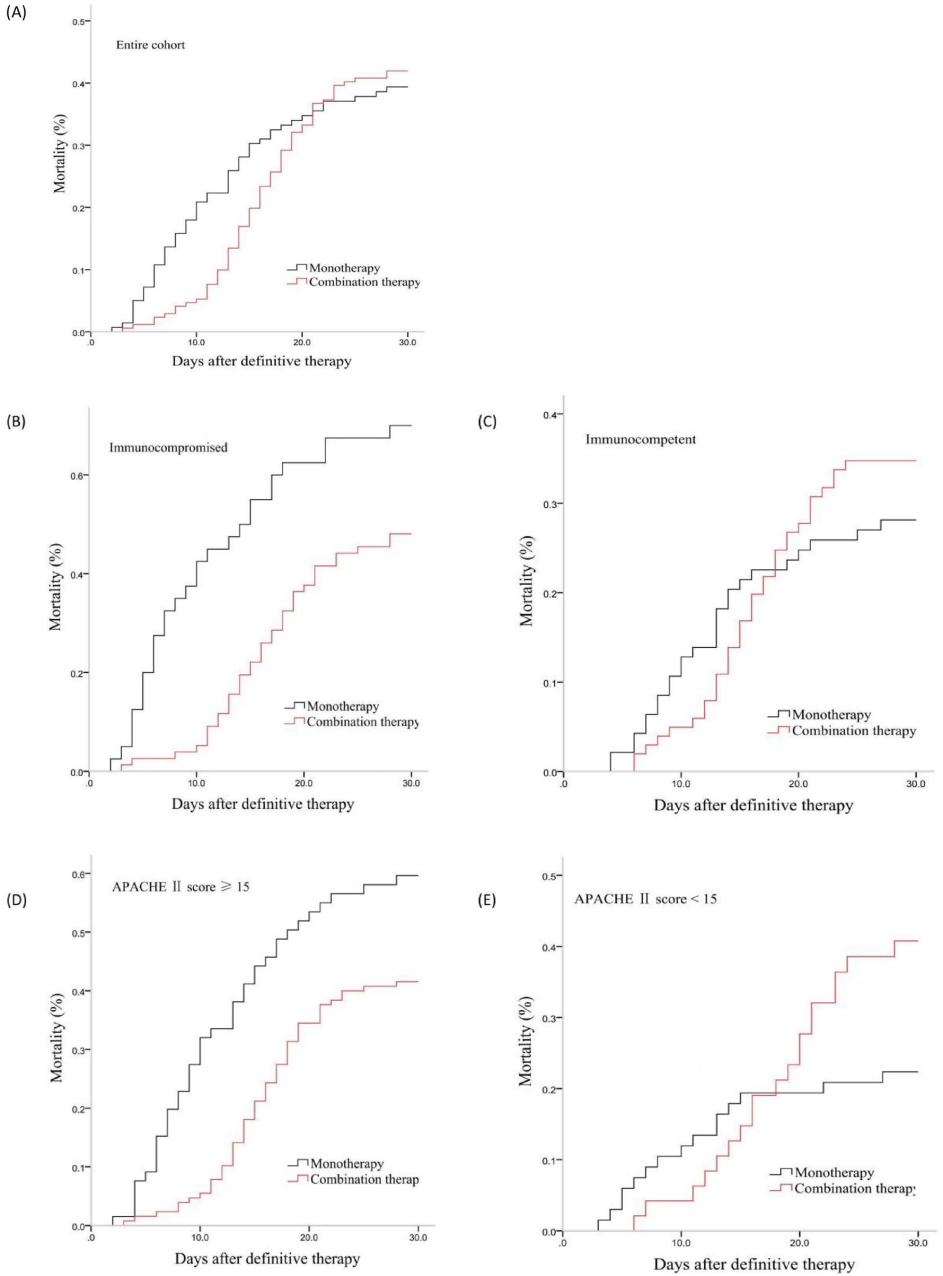
Survival rate of *S. maltophilia*-HAP patients treated with montherapy and combination definitive therapy (censored at 30 d after definitive therapy). Figure legend Cox survival curves demonstrated that combination treatment was related to similar 30-day mortality risk in the overall patient cohort, patients with APACHE II scores < 15, and immunocompetent individuals, but with a decline in 30-day mortality risk among those patients with APACHE II scores ≥ 15 and immunocompromised individuals after controlling for WPS

After controlling for WPS values, a multivariate logistic regression analysis revealed that combination therapy and monotherapy were associated with similar 30-day mortality (*OR* 1.124, *95% CI* 0.707–1.786, *P* = 0.622), clinical response (*OR* 0.987, *95% CI* 0.626–1.557, *P* = 0.956), and microbial eradication rates (*OR* 1.035, *95% CI* 0.640–1.677, *P* = 0.888) in the overall patient cohort (Table 2, Fig. 2 and Fig 3A).

**Table 2:** Impact of different definitive regimens on the outcomes among patients with *S. maltophilia*-HAP via a WPS analysis

Outcome	Patients (*n*, %)		Univariate logistic analysis		Multivariate logistic analysis	
			*OR (95% CI)*	*P*	*OR (95% CI)	*P*
30-day mortality	Entire cohort	126/307 (41.0)	1.104 (0.698–1.747)	0.671	1.124 (0.707–1.786)	0.622
	Immunocompromised	65/117 (55.6)	0.396 (0.176–0.892)	0.025	0.404 (0.170–0.962)	0.041
	Immunocompetent	61/190 (32.1)	1.597 (0.864–2.592)	0.135	1.349 (0.712–2.554)	0.359
	APACHE II score ≥ 15	92/193 (47.7)	0.471 (0.256–0.866)	0.015	0.494 (0.256–0.951)	0.035
	APACHE II score < 15	34/114 (29.8)	2.956 (1.290–6.770)	0.010	2.357 (0.820–6.677)	0.111
30-day clinical response	Entire cohort	167/307 (54.4)	0.999 (0.636–1.570)	0.996	0.987 (0.626–1.557)	0.956
	Immunocompromised	52/117 (44.4)	2.523 (1.121–5.674)	0.025	2.601 (1.088–6.218)	0.032
	Immunocompetent	115/190 (60.5)	0.709 (0.395–1.271)	0.248	0.833 (0.453–1.530)	0.555
	APACHE II score ≥ 15	95/193 (49.2)	2.128 (1.153–3.926)	0.016	2.096 (1.082–4.058)	0.028
	APACHE II score < 15	72/114 (63.2)	0.440 (0.201–0.965)	0.041	0.584 (0.212–1.608)	0.298
30-day microbiology eradiction ǂ	Entire cohort	131/272 (48.2)	1.049 (0.649–1.693)	0.846	1.035 (0.640–1.677)	0.888
	Immunocompromised	38/106 (35.8)	2.625 (1.049–6.569)	0.039	2.788 (1.027–7.567)	0.044
	Immunocompetent	93/166 (56.0)	0.827 (0.448–1.528)	0.544	0.955 (0.504–1.810)	0.887
	APACHE II score ≥ 15	79/175 (45.1)	2.199 (1.150–2.407)	0.017	2.256 (1.123–4.534)	0.022
	APACHE II score < 15	52/97 (53.6)	0.388 (0.168–0.896)	0.027	0.478 (0.164–1.397)	0.177

^*^adjusted by WPS for treatment

ǂ35 patients didn't perform repeated culture of eligible respiratory tract samples

### The association between immune status and patient outcomes following definitive antimicrobial treatment

No differences in 30-day mortality, clinical response, or microbial eradication were observed in immunocompetent patients following administration of the definitive treatment regimen (Table 2). In contrast, after controlling for WPS values, combination therapy was associated with lower odds of 30-day mortality (*OR* 0.404, *95% CI* 0.170–0.962, *P* = 0.041), higher 30-day clinical response rates (*OR* 2.601, *95% CI* 1.088–6.218, *P* = 0.032), and increased 30-day microbial eradication rates (*OR* 2.788, *95% CI* 1.027–7.567, *P* = 0.044) in comparison with monotherapy in immunocompromised patients (Table 2 and Fig 3B-C).

### The association between APACHE II score and definitive antimicrobial treatment outcomes

Univariate analyses indicated that in individuals with APACHE II scores < 15, the combination treatment was associated with a higher 30-day mortality risk, as well as lower odds of 30-day clinical response and microbial eradication (Fig. 3). However, after controlling for WPS values, both combination therapy and monotherapy were found to be associated with similar odds of 30-day mortality (*OR* 2.357, *95% CI* 0.820–6.677, *P* = 0.111), 30-day clinical response (*OR* 0.584, *95% CI* 0.212–1.608, *P* = 0.298), and 30-day microbial eradication (*OR* 0.478, *95% CI* 0.164–1.397, *P* = 0.177) (Table 2).

In patients with APACHE II scores ≥ 15 and after controlling for WPS values, combination therapy was found to be associated with a reduced risk of 30-day mortality (*OR* 0.494*, 95% CI* 0.256–0.951*, P* = 0.035), as well as higher 30-day clinical response (*OR* 2.096*, 95% CI* 1.082–4.058*, P* = 0.028) and microbial eradication rates (*OR* 2.256*, 95% CI* 1.123–4.534*, P* = 0.022), compared with monotherapy (Table 2 and Fig 3D-E)).

### Identification of predictors of patient 30-day mortality

Relative to those patients that remained alive after 30 days, deceased individuals tended to be older (median, 71.5 yrs vs 61.0 yrs, *P* < 0.001) and were more likely to exhibit cardiovascular disease (63.6% vs 52.8%, *P* = 0.022) and immunocompromised status (50.4% vs 29.2%, *P* < 0.001). Deceased patients also exhibited higher APACHE II scores (median, 20.0 vs 15.0, *P* < 0.001), a higher incidence of septic shock (24.0% vs 13.5%, *P* = 0.011), and higher rates of invasive mechanical ventilation (62.1% vs 42.7%, *P* < 0.001), whereas they were less likely to have received appropriate empirical treatment (7.0% vs 15.7%, *P* = 0.027). In addition, the median interval between HAP onset and the initiation of definitive therapy (6.0 days vs 5.0 days, P = 0.004) was also longer among deceased patients relative to survivors (Additional file 1).

Consistent with these results, a multivariate backward stepwise logistic regression analysis revealed that age (*OR* 1.038, *95% CI* 1.020–1.056, *P* < 0.001), immunocompromised status (*OR* 1.846, *95% CI* 1.260–3.158, *P* = 0025), APACHE II score (*OR* 1.076, *95% CI* 1.025–1.130, *P* = 0.003), appropriate empirical treatment (*OR* 0.383, *95% CI* 0.159–0.922, *P* = 0.032), and the interval between HAP onset and definitive treatment (*OR* 1.362, *95% CI* 1.106–1.678, *P* = 0.004) were all independently related to the risk of 30-day all-cause mortality among *S. maltophilia-*HAP patients (Additional file 1)*.*

### The impact of definitive treatment regimens on patient outcomes after controlling for predictors of mortality

Consistent with the results of the propensity score weighting analysis, after controlling for age (continuous variable), immunocompromised status (compromised or not), APACHE II score (continuous variable), appropriate empirical therapy (treated or not), and days from illness onset to definitive therapy (continuous variable), similar 30-day mortality rates were observed for both the combination therapy and monotherapy in the overall patient cohort, immunocompetent patients, and patients with APACHE II scores < 15, together with reduced risks of 30-day mortality in immunocompromised patients and patients with APACHE II scores ≥ 15 (Additional file 1).

### The efficacy of TMP-SMX alone and with the addition of quinolone as antimicrobial regimens on the 30-day mortality of patients with *S. maltophilia*-HAP

After controlling for WPS values, it was found that compared with TMP-SMX alone, the TMP-SMX + quinolone combination regimen was associated with similar risks of 30-day mortality in the entire cohort, immunocompetent patients, and patients with APACHE II scores < 15, and with a decreased risk of 30-day mortality in immunocompromised patients and patients with APACHE II scores ≥ 15 (Additional file 1).

## Discussion

In the present multicenter retrospective cohort study, comprehensive subgroup analyses ultimately led to the identification of multiple predictors of *S. maltophilia-*HAP patient mortality while highlighting the distinct effects of monotherapy and combination therapy regimens in different subsets of patients. Specifically, individuals with severe disease (APACHE II scores ≥ 15) and immunocompromised patients may potentially benefit from combination therapy as compared to monotherapy. In immunocompetent patients and individuals with non-severe disease, however, these two interventional strategies were associated with comparable efficacy outcomes.

In line with prior research [5, 16, 17], the 30-day mortality rate among patients in this study was 41.0%. *S. maltophilia-*HAP patient outcomes can be strongly impacted by patient-specific characteristics, treatment strategies, and associated complications. While this pathogen exhibits relatively limited virulence in the general population, *S. maltophilia* poses a significant risk to patients who are immunocompromised. Indeed, 38.1% of patients in this study cohort were found to be immunocompromised, with immunocompromised status having been identified as an independent predictor of 30-day patient mortality. Even when provided with appropriate antimicrobial therapy, patients who are immunocompromised remain at higher risk of mortality and are likely to remain symptomatic for extended periods as compared to immunocompetent patients [18]. Advanced age has also been shown to be an independent risk factor for pneumonia, likely owing to the link between aging and declines in both organ and overall immune function [19]. Higher APACHE II scores were also found to be related to an elevated mortality rate in this study cohort, consistent with prior research results [20, 21]. For example, Kanchanasuwan et al. [21] and Puech et al. [22] determined that appropriate empirical antimicrobial treatment was related to a significant reduction in 30-day mortality, with higher baseline APACHE II scores serving as an independent predictor of mortality risk. A longer interval between HAP onset and definitive treatment was also associated with a higher risk of death in the present study, highlighting the importance of promptly administering appropriate antimicrobial drugs to most effectively treat *S. maltophilia*-HAP.

Given that *S. maltophilia* isolates generally carry high numbers of antimicrobial resistance genes and associated mutations the selection of appropriate antimicrobial therapies for *S. maltophilia-*HAP patients can be extremely challenging. To date, MIC thresholds for *S. maltophilia* have been established by the CLSI for 7 drugs, including TMP-SMX, ticarcillinclavulanate, ceftazidime, cefiderocol, levofloxacin, minocycline, and chloramphenicol [23]. Ongoing debate remains regarding the relative value of treating *S. maltophilia* with one or more than one antimicrobial drugs [24–26], with some clinicians favoring combination therapy in light of the reported synergistic effects of combining two or more drugs in vitro [27–29]*.* Clinical data regarding the efficacy of these combination regimens, however, is lacking. Guerci et al. [24] conducted a retrospective review of 282 patients infected with *S. maltophilia-*HAP across 25 ICUs in France. While 59.4% of the patients received combination antimicrobial treatment, this was not found to lead to any survival benefits (*HR* 1.27, *95% CI* 0.88; 1.83, *P* = 0.20). Araoka et al. [25] similarly observed no improvement in 30-day mortality for patients treated with a combination of SXT + fluoroquinolone relative to monotherapy (55% vs 33%, *P* = 0.64), despite observing beneficial effects of the combination antimicrobial treatment in in vitro studies. Wafa Ibn Saied et al. also found that adequate treatment, whether monotherapy or a combination of antimicrobials, did not affect mortality in patients with ventilator-associated pneumonia caused by *S. maltophilia* [26]. Consistent with these prior studies, combination therapy herein failed to decrease mortality rates or improve clinical response or microbiologic eradication rates among the overall *S. maltophilia-*HAP patient cohort. When evaluating patients diagnosed with *S. maltophilia* pneumonia, however, Shah et al. [30] observed increased 30-day mortality rates in patients in the combination therapy group compared with the monotherapy group. A meta-analysis conducted by Prawang et al. showed that monotherapy was linked to significantly reduced mortality rates in patients with *S. maltophilia*-HAP infections (*HR* 1.42, *95% CI* 1.04–1.94) compared with combination therapy [31]. Notably, the patients in the two treatment groups in this study were not adequately matched, with those in the combination therapy group having much higher APACHE II scores than those in the monotherapy group (19.0 vs 16.0, *P* = 0.05) [31]. As the authors did not control for confounding effects when assessing mortality outcomes, the conclusion that combination therapy is inferior to monotherapy does not appear to be well supported.

Subgroup analyses performed herein demonstrated that combination treatment was linked to better outcomes among individuals with APACHE II scores ≥ 15 and immunocompromised patients. Muder et al. [32] previously conducted a prospective multicenter observational analysis of 91 cases of *S. maltophilia* bacteremia in immunocompromised patients and observed significantly reduced mortality among individuals administered more than one class of susceptible antimicrobial drugs as compared to those administered just one drug class (11% vs 31%, *P* < 0.05). Combination therapy has also previously been tied to better outcomes among patients with severe infections caused by various pathogens, including *S. maltophilia.* For sample, Latzer et al. [33] conducted a retrospective analysis of 68 critically ill pediatric patients suffering from *S. maltophilia* bacteremia across the four largest Israeli pediatric ICUs, ultimately revealing that treatment with a combination consisting of ciprofloxacin, trimethoprim-sulfamethoxazole, and minocycline was associated with the longest survival duration. In another retrospective multinational analysis, Albasanz-Puig et al. [34] determined that the 7-day case fatality rate of neutropenic patients suffering from Gram-negative bacteria bloodstream infections was lower for patients administered combination therapy. Relative to monotherapy, combination treatment can synergistically kill target pathogens while potentially delaying the emergence of drug resistance during the treatment process. This might be an explanation of why the mortality remained low in patients in the 10 days following the combination definitive therapy. Combination therapy can also contribute to more favorable pharmacokinetics for the administered antimicrobial drugs, particularly among patients who may exhibit enhanced renal clearance such as those with severe disease or immunocompromised individuals, contributing to higher odds of achieving the pharmacokinetic/pharmacodynamic targets [35]. Combination treatment strategies can also overcome reductions in antimicrobial efficacy resulting from toxicity-related limitations to single-agent drug dosing [36]. Relative to randomized controlled trials (RCTs), observational studies are more prone to selection bias, and a range of confounding factors may have influenced the results of this study [37]. For example, in individuals with APACHE II scores < 15, the combination regimen was associated with an increased risk of 30-day mortality risk, as shown by the univariate analysis. However, there was a clear imbalance between the two groups, e.g., the patients receiving combination therapy were older (median: 66.0 years vs 59.1 years, *p* = 0.024), more patients had diabetes mellitus (51.2% vs 28.2%, *p* = 0.014), and more patients developed septic shock (16.3% vs 4.2%, *p* = 0.027). After controlling for these confounders, no differences were observed between patients treated with monotherapy or combination therapy. To better account for these effects, two different approaches were employed to control for them, confirming the relationship between these antimicrobial regimens and *S. maltophilia-*HAP patient clinical outcomes.

This study is subject to some limitations. For one, as a retrospective analysis, these results are potentially susceptible to selection and recall bias. Other factors may have also impacted the relative efficacy of the selected antimicrobial treatment regimens such as antibiotics class, dosing, or duration of effusion [38, 39]. The potential effects of these variables on the study conclusions could not be assessed given the relatively limited study sample size, which precluded detailed subgroup analyses. The retrospective nature of this study also prevented any reliable analysis of antibiotic toxicity. While treatment failure can also result from heteroresistance [40], as this was a retrospective analysis it was similarly impossible to isolate pathogens for heteroresistance testing. Finally, this study did not include certain novel antimicrobial agents such as cefiderocol [41], and clinical validation will thus be necessary to clarify the relative value of these drugs when treating *S. maltophilia-*HAP.

In summary, the present results suggest that combination therapy may be of value when treating *S. maltophilia-*HAP patients who are immunocompromised or exhibit APACHE II scores ≥ 15, providing a potential avenue towards improving outcomes for these patients. However, future large-scale RCTs will be vital to validate these findings and clarify their clinical relevance.

## Supplementary Information

**Additional file 1.** Table S1,2,3...7.

## Data Availability

The datasets used and/or analyzed during the current study are available from the corresponding author on reasonable request.

## Abbreviations

HAP: Hospital-acquired pneumonia
APACHE: Acute Physiology and Chronic Health Evaluation
GNB: Gram-negative bacteria
NFGNB: Non-fermenting Gram-negative bacilli
MDROs: Multidrug resistant organisms
TMP-SMX: Trimethoprim/sulfamethoxazole
TGC: Tigecycline
MIC: Minimum inhibitory concentration
PSB: Protected specimen brush
BALF: Bronchoalveolar lavage fluid
BMI: Body mass index
CRO: Carbapenem-resistant organisms
ICU: Intensive care unit

## References

[1] Millar BC, McCaughan J, Rendall JC, Moore JE (2023). Infection dynamics of *Stenotrophomonas maltophilia* in patients with cystic fibrosis. J Infect.

[2] Fluit AC, Bayjanov JR, Aguilar MD, Canton R, Elborn S, Tunney MM (2022). Taxonomic position, antibiotic resistance and virulence factor production by *Stenotrophomonas* isolates from patients with cystic fibrosis and other chronic respiratory infections. BMC Microbiol.

[3] Hafiz TA, Aldawood E, Albloshi A, Alghamdi SS, Mubaraki MA, Alyami AS (2022). *Stenotrophomonas maltophilia* epidemiology, resistance characteristics, and clinical outcomes: understanding of the recent three years' trends. Microorganisms.

[4] Trifonova A, Strateva T (2019). *Stenotrophomonas maltophilia*—a low-grade pathogen with numerous virulence factors. Infect Dis (Lond).

[5] Wang Y, Wang Y, Rong H, Guo Z, Xu J, Huang X (2022). Risk factors of lower respiratory tract infection caused by *Stenotrophomonas maltophilia*: systematic review and meta-analysis. Front Public Health.

[6] Montoya-Hinojosa EI, Salazar-Sesatty HA, Alvizo-Baez CA, Terrazas-Armendariz LD, Luna-Cruz IE, Alcocer-Gonzalez JM (2023). Antibiofilm and antimicrobial activity of curcumin-chitosan nanocomplexes and trimethoprim-sulfamethoxazole on *Achromobacter*, *Burkholderia*, and *Stenotrophomonas* isolates. Expert Rev Anti Infect Ther.

[7] McCutcheon JG, Dennis JJ (2021). The potential of phage therapy against the emerging opportunistic pathogen *Stenotrophomonas maltophilia*. Viruses.

[8] Brooke JS (2021). Advances in the microbiology of *Stenotrophomonas maltophilia*. Clin Microbiol Rev..

[9] Jian J, Xie Z, Chen L (2022). Risk factors for mortality in hospitalized patients with *Stenotrophomonas maltophilia* bacteremia. Infect Drug Resist.

[10] Vouillamoz J, Moreillon P, Giddey M, Entenza JM (2008). In vitro activities of tigecycline combined with other antimicrobials against multiresistant gram-positive and gram-negative pathogens. J Antimicrob Chemother.

[11] Kalil AC, Metersky ML, Klompas M, Muscedere J, Sweeney DA, Palmer LB (2016). Management of adults with hospital-acquired and ventilator-associated pneumonia: 2016 Clinical practice guidelines by the infectious diseases society of America and the American Thoracic Society. Clin Infect Dis.

[12] Ramirez JA, Musher DM, Evans SE, Dela Cruz C, Crothers KA, Hage CA (2020). Treatment of community-acquired pneumonia in immunocompromised adults: a consensus statement regarding initial strategies. Chest.

[13] Singer M, Deutschman CS, Seymour CW, Shankar-Hari M, Annane D, Bauer M (2016). The Third International Consensus definitions for sepsis and septic shock (Sepsis-3). JAMA.

[14] Chen L, Han X, Li Y, Li M (2021). Assessment of mortality-related risk factors and effective antimicrobial regimens for treatment of bloodstream infections caused by carbapenem-resistant Enterobacterales. Antimicrob Agents Chemother..

[15] Muthuri SG, Venkatesan S, Myles PR, Leonardi-Bee J, Al Khuwaitir TS, Al Mamun A (2014). Effectiveness of neuraminidase inhibitors in reducing mortality in patients admitted to hospital with influenza A H1N1pdm09 virus infection: a meta-analysis of individual participant data. Lancet Respir Med.

[16] Insuwanno W, Kiratisin P, Jitmuang A (2020). *Stenotrophomonas maltophilia* infections: clinical characteristics and factors associated with mortality of hospitalized patients. Infect Drug Resist.

[17] Alsuhaibani M, Aljarbou A, Althawadi S, Alsweed A, Al-Hajjar S (2021). *Stenotrophomonas maltophilia* bacteremia in children: risk factors and mortality rate. Antimicrob Resist Infect Control.

[18] Dropulic LK, Lederman HM (2016). Overview of infections in the immunocompromised host. Microbiol Spectr.

[19] Hespanhol V, Barbara C (2020). Pneumonia mortality, comorbidities matter?. Pulmonology.

[20] Insuwanno W, Kiratisin P, Jitmuang A (2020). *Stenotrophomonas maltophilia* infections: clinical characteristics and factors associated with mortality of hospitalized patients. Infect Drug Resist..

[21] Kanchanasuwan S, Rongmuang J, Siripaitoon P, Kositpantawong N, Charoenmak B, Hortiwakul T (2022). Clinical characteristics, outcomes, and risk factors for mortality in patients with *Stenotrophomonas maltophilia* bacteremia. J Clin Med.

[22] Puech B, Canivet C, Teysseyre L, Miltgen G, Aujoulat T, Caron M (2021). Effect of antibiotic therapy on the prognosis of ventilator-associated pneumonia caused by *Stenotrophomonas maltophilia*. Ann Intensive Care.

[23] Tamma PD, Aitken SL, Bonomo RA, Mathers AJ, van Duin D, Clancy CJ (2022). Infectious diseases society of America guidance on the treatment of AmpC beta-lactamase-producing Enterobacterales, Carbapenem-resistant *Acinetobacter baumannii*, and *Stenotrophomonas maltophilia* infections. Clin Infect Dis.

[24] Guerci P, Bellut H, Mokhtari M, Gaudefroy J, Mongardon N, Charpentier C (2019). Outcomes of Stenotrophomonas maltophilia hospital-acquired pneumonia in intensive care unit: a nationwide retrospective study. Crit Care.

[25] Araoka H, Baba M, Okada C, Abe M, Kimura M, Yoneyama A (2017). Evaluation of trimethoprim-sulfamethoxazole based combination therapy against *Stenotrophomonas maltophilia*: in vitro effects and clinical efficacy in cancer patients. Int J Infect Dis.

[26] Ibn Saied W, Merceron S, Schwebel C, Le Monnier A, Oziel J, Garrouste-Orgeas M (2020). Ventilator-associated pneumonia due to *Stenotrophomonas maltophilia*: risk factors and outcome. J Infect.

[27] Wei C, Ni W, Cai X, Zhao J, Cui J (2016). Evaluation of Trimethoprim/Sulfamethoxazole (SXT), Minocycline, Tigecycline, Moxifloxacin, and Ceftazidime alone and in combinations for SXT-Susceptible and SXT-resistant *Stenotrophomonas maltophilia* by in vitro time-kill experiments. PLoS One..

[28] Chumbita M, Puerta-Alcalde P, Gudiol C, Garcia-Pouton N, Laporte-Amargos J, Ladino A (2022). Impact of empirical antibiotic regimens on mortality in neutropenic patients with bloodstream infection presenting with septic shock. Antimicrob Agents Chemother..

[29] Band VI, Weiss DS (2019). Heteroresistance: a cause of unexplained antibiotic treatment failure?. PLoS Pathog..

[30] Shah MD, Coe KE, El Boghdadly Z, Wardlow LC, Dela-Pena JC, Stevenson KB (2019). Efficacy of combination therapy versus monotherapy in the treatment of *Stenotrophomonas maltophilia* pneumonia. J Antimicrob Chemother.

[31] Prawang A, Chanjamlong N, Rungwara W (2022). Combination therapy versus monotherapy in the treatment of *Stenotrophomonas maltophilia* infections: a systematic review and meta-analysis. Antibiotics.

[32] Muder RR, Harris AP, Muller S, Edmond M, Chow JW, Papadakis K (1996). Bacteremia due to *Stenotrophomonas* (Xanthomonas) *maltophilia*: a prospective, multicenter study of 91 episodes. Clin Infect Dis.

[33] Tokatly Latzer I, Nahum E, Cavari Y, Lazar I, Ben-Ari Y, Ben-Shimol S (2019). Treatment outcomes of *Stenotrophomonas maltophilia* bacteremia in critically Ill children: a multicenter experience. Pediatr Crit Care Med.

[34] Albasanz-Puig A, Gudiol C, Puerta-Alcalde P, Ayaz CM, Machado M, Herrera F (2021). Impact of the inclusion of an aminoglycoside to the initial empirical antibiotic therapy for gram-negative bloodstream infections in hematological neutropenic patients: a propensity-matched cohort study (AMINOLACTAM study). Antimicrob Agents Chemother..

[35] Cook AM, Hatton-Kolpek J (2019). Augmented renal clearance. Pharmacotherapy.

[36] Carrick S, Parker S, Thornton CE, Ghersi D, Simes J, Wilcken N (2009). Single agent versus combination chemotherapy for metastatic breast cancer. Cochrane Database Syst Rev..

[37] Deeks JJ, Dinnes J, D'Amico R, Sowden AJ, Sakarovitch C, Song F (2003). Evaluating non-randomised intervention studies. Health Technol Assess..

[38] Zha L, Zhang D, Pan L, Ren Z, Li X, Zou Y (2021). Tigecycline in the treatment of ventilator-associated pneumonia due to *Stenotrophomonas maltophilia*: a multicenter retrospective cohort study. Infect Dis Ther.

[39] Junco SJ, Bowman MC, Turner RB (2021). Clinical outcomes of *Stenotrophomonas maltophilia* infection treated with trimethoprim/sulfamethoxazole, minocycline, or fluoroquinolone monotherapy. Int J Antimicrob Agents..

[40] Martinez-Servat S, Yero D, Huedo P, Marquez R, Molina G, Daura X (2018). Heterogeneous colistin-resistance phenotypes coexisting in *Stenotrophomonas maltophilia* isolates influence colistin susceptibility testing. Front Microbiol.

[41] McCreary EK, Heil EL, Tamma PD (2021). New perspectives on antimicrobial agents: Cefiderocol. Antimicrob Agents Chemother..

