# Supplementary material for: Assessment of the relative benefits of monotherapy and combination therapy approaches to the treatment of hospital-acquired Stenotrophomonas maltophilia pneumonia: a multicenter, observational, real-world study
Source: Ann Intensive Care. 2023 Jun 6;13:47. doi: 10.1186/s13613-023-01144-7 (PMC10244312; doi:10.1186/s13613-023-01144-7)
Supplement: Supplementary file 1 — Additional file 1. Table S1,2,3...7. [file 13613_2023_1144_MOESM1_ESM.docx]

**Additional Material 1: Details of participating centers**

| **Name of the hospital** | **Province, city** | **Teaching Hospital** | **Beds** | **Staffs of Clinical Microbioloy Lab (*n*)** |
| --- | --- | --- | --- | --- |
| Nanjing Lishui people’s Hospital | Jiangsu, Nanjing | Yes | 1000 | 8 |
| Jiangsu Province Hospital | Jiangsu, Nanjing | Yes | 5200 | 25 |
| Liyang People’s Hospital, | Jiangsu, Liyang | Yes | 1000 | 8 |
| Beijing Jishuitan Hospital | Beijing | Yes | 2600 | 15 |

**Additional Material 2 Definition of underlying diseases**

1. Cardiovascular disease included coronary heart disease and chronic congestive heart failure;
2. Coronary heart disease included angina pectoris, myocardial infarction, ischemic cardiomyopathy;
3. Heart failure was defined as a clinical syndrome consisting of dyspnea, malaise, swelling and/or decreased exercise capacity due to the loss of compensation for cardiac pumping function due to structural and/or functional abnormalities of the heart.
4. Chronic obstructive pulmonary disease was defined as: persistent airflow limitation, FEV_1_ / FVC < 70% post bronchodilator;
5. Cerebrovascular diseases included transient ischemic attack, cerebral hemorrhage, subarachnoid hemorrhage, cerebral infarction;
6. Diabetes mellitus: included diabetes mellitus type 1 and diabetes mellitus type 2, not included impaired glucose tolerance and impaired fasting glycaemia;
7. Chronic kidney disease included diabetic nephropathy, hypertensive renal damage, chronic glomerulonephritis, chronic pyelonephritis, lupus nephritis, IgA nephropathy, nephrotic syndrome, hereditary kidney disease;
8. Immunocompromised status included primary immune deficiency diseases, active malignancy, HIV infection with a CD4 T-lymphocyte count < 200 cells/mL or percentage < 14%, immunosuppressive therapy, solid organ transplantation, hematopoietic stem cell transplantation, splenectomy [1];
9. Immunosuppressive therapy: was defined as receiving cancer chemotherapy, receiving corticosteroid therapy with a dose ≥ 20 mg prednisone or equivalent daily for ≥ 14 d or a cumulative dose > 600 mg of prednisone, receiving biological immune modulators, receiving disease-modifying antirheumatic drugs or other immunosuppressive drugs (eg, cyclosporin, cyclophosphamide, hydroxychloroquine, methotrexate);
10. Septic shock was diagnosed when vasopressors are required to maintain mean arterial pressure ≥ 65 mmHg and serum lactate is ≥ 2 mmol/L despite adequate volume resuscitation [2].
11. Ramirez JA, Musher DM, Evans SE, Dela Cruz C, Crothers KA, Hage CA, et al. Treatment of Community-Acquired Pneumonia in Immunocompromised Adults: A Consensus Statement Regarding Initial Strategies. Chest. 2020;158(5):1896-1911.
12. Singer M., Deutschman C.S., Seymour C.W., Shankar-Hari M., Annane D., Bauer M., Bellomo R., Bernard G.R., Chiche J.-D., Coopersmith C.M., et al. The Third International Consensus Definitions for Sepsis and Septic Shock (Sepsis-3) JAMA. 2016;315:801–810.

**Additional Material 3 Detailed information of definitive antimcrobial regimens**

| **Antimicrobial regimen** | **Cases (*n*, %)** |
| --- | --- |
| Monotherapy | 136 (44.3) |
| TMP-SMX | 52 (16.9) |
| Levofloxacin | 44 (14.3) |
| Moxifloxacin | 29 (9.4) |
| Tigecycline | 6 (2.0) |
| Minocycline | 3 (1.0) |
| Ceftazidime | 2 (0.7) |
| Combination therapy | 171 (55.7) |
| TMP-SMX + levofloxacin | 53 (17.3) |
| TMP-SMX + moxifloxacin | 42 (13.7) |
| Levofloxacin + tigecycline | 7 (2.3) |
| Levofloxacin + minocycline | 2 (0.7) |
| Moxifloxacin + tigecycline | 3 (1.0) |
| Moxifloxacin + minocycline | 1 (0.3) |
| TMP-SMX + levofloxacin + tigecycline | 10 (3.3) |
| TMP-SMX + levofloxacin + minocycline | 3 (1.0) |
| TMP-SMX + moxifloxacin + tigecycline | 13 (4.2) |
| TMP-SMX + moxifloxacin + minocycline | 1 (0.3) |
| TMP-SMX + levofloxacin + ceftazidime | 12 (3.9) |
| TMP-SMX + moxifloxacin + ceftazidime | 13 (4.2) |
| TMP-SMX + tigecycline + ceftazidime | 6 (2.0) |

**Additional Material 4 Comparison of baseline clinical characteristics and management between survival and deceased patients with S. maltophilia-HAP**

| **Variable** | **Deceased**  **(*n* = 126)** | **Survival**  **(*n* = 181)** | ***P* value** |
| --- | --- | --- | --- |
| Age (years, median, IQR) ^#^ | 71.5 (60.0, 81.0) | 61.0 (51.0, 70.0) | **< 0.001** |
| Male (*n*, %) | 97 (77.0) | 135 (74.6) | 0.630 |
| BMI (kg/m^2^, mean ± SD) | 22.8 ± 2.7 | 24.4 ± 10.6 | 0.115 |
| Participating hospital |  |  |  |
| 1 | 43 (34.1) | 62 (34.3) | 0.785 |
| 2 | 29 (23.0) | 52 (28.7) | 0.186 |
| 3 | 21 (16.7) | 33 (18.2) | 0.608 |
| 4 | 33 (26.2) | 34 (18.8) | 0.175 |
| Comorbidities (*n*, %) |  |  |  |
| Cardiovascular disease ^#^ | 82 (65.0) | 94 (51.9) | **0.022** |
| Diabetes mellitus | 47 (37.3) | 66 (36.5) | 0.881 |
| Cerebrovascular disease | 43 (34.1) | 48 (26.5) | 0.151 |
| Chronic kidney disease | 23 (18.3) | 32 (17.7) | 0.897 |
| COPD | 25 (19.8) | 27 (14.9) | 0.258 |
| Chronic liver disease | 9 (7.1) | 18 (9.9) | 0.394 |
| Asthma | 9 (7.1) | 15 (8.3) | 0.713 |
| Immunocompromised status ^#^ | 65 (51.6) | 52 (28.7) | < **0.001** |
| Baseline clinical features and severity |  |  |  |
| Leukocyte counts (×10^9^/L) | 10.3 ± 4.6 | 11.6 ± 9.5 | 0.203 |
| PCT > 2 ng/dL (*n*, %) | 44 (34.9) | 66 (36.5) | 0.781 |
| PO_2_/FiO_2_ < 300 mmHg (*n*, %) | 23 (18.3) | 21 (11.6) | 0.102 |
| APACHE Ⅱ score (median, IQR) ^#^ | 20.0 (14.0, 24.0) | 15.0 (12.0, 21.0) | < **0.001** |
| Coinfection (*n*, %) | 65 (51.6) | 85 (47.0) | 0.425 |
| With other CRO (*n*, %) | 30 (23.8) | 46 (25.4) | 0.749 |
| Complications and Management |  |  |  |
| Secondary bacteremia (*n*, %) | 7 (5.6) | 7 (3.9) | 0.486 |
| Septic shock (*n*, %) ^#^ | 31 (24.6) | 24 (13.3) | **0.011** |
| Appropriate empirical therapy (*n*, %) ^#^ | 9 (7.1) | 28 (15.5) | **0.027** |
| Noninvasive mechanical ventilation (*n*, %) | 87 (69.0) | 115 (63.5) | 0.317 |
| Invasive mechanical ventilation (*n*, %) ^#^ | 79 (62.9) | 76 (42.0) | < **0.001** |
| Vasopressor use (*n*, %) | 8 (6.3) | 10 (5.5) | 0.762 |
| ICU admission (*n*, %) | 98 (77.8) | 135 (74.6) | 0.520 |
| Days from illness onset to definitive therapy (median, IQR) ^#^ | 6.0 (4.8, 6.0) | 5.0 (4.0, 6.0) | **0.004** |
| Outcomes |  |  |  |
| 30-day clinical response | 6 (4.8) | 161 (89.0) | < **0.001** |
| 30-day microbiology eradiction | 3/120 (2.5) | 128/152 (84.2) | < **0.001** |

IQR: interquartile range; SD: standard deviation; COPD: chronic obstructive pulmonary disease. BMI: body mass index; PCT: procalcitonin; PO_2_/FiO_2_: arterial pressure of oxygen/fraction of inspiration oxygen; APACHE: Acute Physiology and Chronic Health Evaluation; CRO: carbapenem-resistant organism; ICU: intensive care unit. HAP: hospital-acquired pneumonia. Immunocompromised status included primary immune deficiency diseases, active malignancy, HIV infection with a CD4 T-lymphocyte count < 200 cells/mL or percentage < 14%, immunosuppressive therapy, solid organ transplantation, hematopoietic stem cell transplantation, splenectomy. The bolded values are p-values < 0.05, which represented significant differences between deceased and survival patients. ^#^: variables cited in the table above were the candidates which were entered into the multivariate logistic regression model.

**Additional Material 5 Risk factors for 30-day mortality in patients with *S. maltophilia*-HAP**

| **Variable** | ***OR (95% CI)*** | ***P* value** |
| --- | --- | --- |
| Age | 1.038 (1.020-1.056) | < 0.001 |
| Immunocompromised status | 1.846 (1.260-3.158) | 0.025 |
| APACHE Ⅱ score | 1.076 (1.025-1.130) | 0.003 |
| Appropriate empirical therapy | 0.383 (0.159-0.922) | 0.032 |
| Days from illness onset to definitive therapy | 1.362 (1.106-1.678) | 0.004 |

OR: Odd ratio; CI: confidence interval.

**Additional Material 6 Impact of different definitive regimens on the outcomes among patients with *S. maltophilia*-HAP after control for predictors of mortality**

| **Outcome** | **Patients (*n*, %)** | | **Multivariate logistic analysis** | |
| --- | --- | --- | --- | --- |
|  |  |  | ****OR (95% CI)*** | ***P*** |
| 30-day mortality | Entire cohort | 126/307 (41.0) | 0.579 (0.331-1.013) | 0.055 |
|  | Immunocompromised | 65/117 (55.6) | 0.158 (0.053-0.473) | 0.001 |
|  | Immunocompetent | 61/190 (32.1) | 1.030 (0.506-2.094) | 0.936 |
|  | APACHE Ⅱ score ≥ 15 | 92/193 (47.7) | 0.264 (0.126-0.554) | < 0.001 |
|  | APACHE Ⅱ score < 15 | 34/114 (29.8) | 2.454 (0.933-6.457) | 0.069 |
| 30-day clinical response | Entire cohort | 167/307 (54.4) | 1.582 (0.925-2.706) | 0.094 |
|  | Immunocompromised | 52/117 (44.4) | 5.874 (1.970-17.515) | 0.001 |
|  | Immunocompetent | 115/190 (60.5) | 0.917 (0.475-1.771) | 0.796 |
|  | APACHE Ⅱ score ≥ 15 | 95/193 (49.2) | 3.727 (1.771-7.839) | 0.001 |
|  | APACHE Ⅱ score < 15 | 72/114 (63.2) | 0.536 (0.231-1.243) | 0.146 |
| 30-day microbiology eradiction ǂ | Entire cohort | 131/272 (48.2) | 1.747 (0.996-3.066) | 0.052 |
|  | Immunocompromised | 38/106 (35.8) | 5.538 (1.601-15.854) | 0.006 |
|  | Immunocompetent | 93/166 (56.0) | 1.062 (0.535-2.109) | 0.864 |
|  | APACHE Ⅱ score ≥ 15 | 79/175 (45.1) | 4.534 (2.015-10.202) | < 0.001 |
|  | APACHE Ⅱ score < 15 | 52/97 (53.6) | 0.441 (0.177-1.098) | 0.078 |

*: adjusted by age (continuous variable), immunocompromised status (yes or not), APACH Ⅱ score (continuous variable), appropriate empirical therapy (yes or not) and days from illness onset to definitive therapy (continuous variable). ǂ: 35 patients did not perform repeated culture from repiratory tract samples.

**Additional file 7 Efficacy of TMP-SMX alone and quinolone-adding antimicrobials regimen on the 30-day mortality of patients with *S. maltophilia*-HAP via a WPS analysis**

| **Patients** | **Regimens** | **30-day mortality** | | |
| --- | --- | --- | --- | --- |
|  |  | **Cases (*n*,%)** | ****OR (95% CI)*** | ***P*** |
| Entire cohort | TMP-SMX | 22/52 (42.3) | *ref* |  |
|  | TMP-SMX + Quinolone | 42/95 (44.2) | 1.080 (0.546-2.140) | 0.824 |
| Immunocompromised | TMP-SMX | 11/15 (73.3) | *ref* |  |
|  | TMP-SMX + Quinolone | 21/39 (53.8) | 0.073 (0.008-0.684) | 0.022 |
| Immunocompetent | TMP-SMX | 11/37 (29.7) | *ref* |  |
|  | TMP-SMX + Quinolone | 21/56 (37.5) | 1.217 (0.485-3.058) | 0.675 |
| APACHE Ⅱ score ≥ 15 | TMP-SMX | 16/24 (66.7) | *ref* |  |
|  | TMP-SMX + Quinolone | 34/75 (45.3) | 0.234 (0.071-0.766) | 0.016 |
| APACHE Ⅱ score < 15 | TMP-SMX | 6/28 (21.4) | *ref* |  |
|  | TMP-SMX + Quinolone | 8/20 (40.0) | 1.698 (0.304-9.470) | 0.546 |

*: adjusted by WPS for treatment.
